# Fetuin-A and Its Association with Anthropometric, Atherogenic, and Biochemical Parameters and Indices among Women with Polycystic Ovary Syndrome

**DOI:** 10.3390/nu14194034

**Published:** 2022-09-28

**Authors:** Karolina Kulik-Kupka, Marzena Jabczyk, Justyna Nowak, Paweł Jagielski, Bartosz Hudzik, Barbara Zubelewicz-Szkodzińska

**Affiliations:** 1 Department of Nutrition-Related Disease Prevention, Department of Metabolic Disease Prevention, Faculty of Health Sciences in Bytom, Medical University of Silesia, 41-900 Bytom, Poland; 2 Department of Cardiovascular Disease Prevention, Department of Metabolic Disease Prevention, Faculty of Health Sciences in Bytom, Medical University of Silesia, 41-900 Bytom, Poland; 3 Department of Nutrition and Drug Research, Institute of Public Health, Faculty of Health Sciences, Jagiellonian University Medical College, 31-066 Cracow, Poland; 4 Third Department of Cardiology, Silesian Center for Heart Disease, Faculty of Medical Sciences in Zabrze, Medical University of Silesia, 41-800 Zabrze, Poland; 5 Department of Endocrinology, District Hospital, 41-940 Piekary Slaskie, Poland

**Keywords:** fetuin-A, polycystic ovary syndrome, dyslipidemia, dysglycemia, anthropometric indices, BAI, VAI, ABSI, LAP, AIP, atherogenic coefficient, Castelli risk index-I, Castelli risk index-II

## Abstract

Background: Polycystic ovary syndrome (PCOS) contributes to metabolic and endocrine complications for women of reproductive age. We set out to assess the relationship between fetuin-A and anthropometric parameters, anthropometric indices, body composition, and atherogenic indices, as well as carbohydrate and lipid profile in women with polycystic ovary syndrome (PCOS). Methods. The study included 49 women with PCOS, aged between 18 and 39 years. All patients were tested for fetuin-A, fasting glucose and insulin, and lipid parameters, after oral-glucose administration were done. All of them underwent anthropometric measurements and body composition analyses such as BMI (Body Mass Index), WHR (Waist to Hip Ratio), WHtR (Waist to Height Ratio), BAI (Body Adiposity Index), VAI (Visceral Adiposity Index), LAP (Lipid Accumulation Product), BRI (Body Roundness Index), ABSI (A Body Shape Index), ABSI z-core (ABSI with added mortality risk in correlation with age and gender), AIP (Atherogenic Risk of Plasma), AC (Atherogenic Coefficient), Castelli risk index-I, and Castelli risk index-II. Results: Obesity was diagnosed in 18 patients (36.7%) based on BMI index and 7 patients (14.3%) based on BAI index. Significantly increased risk of metabolic complications was observed among 26 patients (53.1%) based on waist circumferences. Based on VAI index, risk of metabolic disease was observed among 17 women (34.7%). Dyslipidemia such as hypercholesterolemia, hypertriglyceridemia, and mixed hyperlipidemia was detected among 14 patients (28.6%), and insulin resistance was observed among 29 (59.2%). There was a positive correlation between fetuin-A and total cholesterol (r = 0.30, *p* = 0.0034). There was no statistically significant correlation between fetuin-A and all of the anthropometric measurements and anthropometric indices, atherogenic indices, and other biochemical parameters. Conclusion: Fetuin-A correlates with hypercholesterolemia. It is necessary to conduct further research regarding the relationship between fetuin-A concentrations and body composition, anthropometric indices, and metabolic disorders in women with PCOS. Surprisingly, the effects of concentration of fetuin-A and anthropometric indices (BAI, VAI, LAP, ABSI, ABSI z-core) in woman with PCOS have not been closely examined. Future studies that take these variables into account will need to be undertaken. More information on the relationship between fetuin-A concentrations and anthropometric indices would aid us in establishing a greater degree of accuracy on this matter.

## 1. Introduction

Polycystic ovary syndrome (PCOS) is a condition of the endocrine system that affects 5–10% of women of childbearing age. Approximately 7–8% of women of reproductive age are affected by PCOS, which is considered the most common cause of female infertility [1]. The diagnosis of PCOS is still a matter of debate. Several concepts of PCOS, metabolic and hormonal, have been considered. Its phenotype is heterogenous, and the most recent diagnostic criteria do not include any indicators that would reflect metabolic dysregulation [2].

Women with PCOS oftentimes suffer from other concomitant disorders such as dyslipidemia, hypertension, chronic inflammation, and obesity. Moreover, insulin resistance (IR) is present in up to 70% of PCOS women [2]. In several human studies, it has been found that altered (increased or decreased) or unchanged circulating fetuin-A levels in obese patients appear to be closely linked to metabolic syndrome (MetS), non-alcoholic fatty liver disease (NAFLD), cardiovascular disease (CVD), and PCOS. Furthermore, there has also been reported an association with impaired glucose intolerance and IR [2,3,4,5]. Although hyperinsulinemia and IR are associated with PCOS pathogenesis, it is not a criterion in PCOS definition. Moreover, those conditions are not seen in all cases [6]. Fetuin-A directly interacts with the insulin receptor and alters insulin signaling via a wide variety of mechanisms, including disruption of insulin-stimulated phosphorylation of the insulin receptor and insulin receptor substrate-1 [7]. Visceral obesity enhances the risk of IR, increases fatty acids synthesis, increases the rate of type 2 diabetes mellitus, and causes cardiovascular complications [2,8,9,10]. Android adipose tissue distribution may also be associated with hyperandrogenemia and decreased concentration of sex hormone binding globulin (SHBG) [11]. This is why PCOS patients have also been shown to have an increased risk of metabolic syndrome [12]. Moreover, it has been reported that increased androgen levels enhance the release of fetuin-A via androgen receptors (AR) in liver cells [6]. Increased androgen levels may affect fetuin-A levels in PCOS. Therefore, elevated fetuin-A levels, by playing a role in many inflammatory processes and hyperandrogenemia, may increase the risk of IR [6].

Recently, many studies have tried to explain the possible mechanisms of metabolic disorders, which is why we look for fetuin-A secretion in PCOS women.

Fetuin-A is a 64 kDa glycoprotein produced in the liver, adipose tissue, and the placenta. It acts as an inhibitor of insulin action through the inhibition of auto-phosphorylation of insulin receptor tyrosine kinase and glucose transporter 4 (GLUT4), causing development of diabetes, insulin resistance, metabolic syndrome, and cardiovascular disease (CVD) [13]. Fetuin-A is a mediator involved in the regulation of calcium metabolism, through reducing and suppressing systemic calcification [14,15]. Decreased serum fetuin levels have been linked to stiffening and calcification of the artery walls. CVDs have become the leading causes of mortality worldwide. Due to the fact that fetuin-A constitutes as one of the most involved inhibitors of calcification, it may be a predictive factor for cardiovascular disease [14]. Notably, low serum fetuin-A levels are linked to ectopic calcification, CVD, chronic lymphocytic leukemia, malnutrition, inflammation, and sepsis, whereas increased levels of fetuin-A play an anti-inflammatory role through inhibiting the secretion of proinflammatory mediators in macrophages [16]. Hence, fetuin-A exhibits atherogenic effects in metabolic syndrome and has been described as significant in the protection against atherosclerotic calcification [14,17]. In contrast, elevated circulating concentrations of fetuin-A have been shown to be related with PCOS, NAFLD, obesity, IR, T2DM, aging, and severity of psoriasis [16]. Moreover, fetuin-A also exerts the features of adipokine and hepatokine, whereas serum paraoxonase-1 (PON-1) enzyme is associated with antioxidant high-density lipoprotein (HDL). The PON-1 gene is expressed mainly in the liver. Decreased serum PON-1 levels may lead to IR, caused by PCOS [18,19].

There are two main sources of fetuin-A: subcutaneous adipose and visceral tissue [20]. It has been stated that visceral tissue secretes a higher amount of fetuin-A, and this mechanism of secretion is more sensitive to nutritional and physiological alterations [20,21]. As mentioned in the literature review [20], several nutrients may alter fetuin-A release. The authors have reported that fatty acids, dietary energy restrictions, exercise, and fasting induce the adipocyte-fetuin-A gene and protein expression, which result is increased secretion of fetuin-A [20]. It has also been shown to stimulate the synthesis of proinflammatory cytokines by macrophages and adipocytes. Therefore, it is regarded as both hepatokine and adipokine [21].

Several studies have found that sometimes adiponectin concentration is lower in women with PCOS compared with the control group matched for BMI. It is believed that fat cell metabolism is associated with ovarian steroidogenesis. Adiponectin pathway disturbance may affect the onset of hyperandrogenism in women with PCOS [22]. Studies comparing associations of fetuin-A concentrations in PCOS are conflicting [23,24,25]. Moreover, the abdominal fat that can express and secrete the adipokine and hepatokine fetuin-A [18] may have a stronger association with morbidity than adiposity [25]. However, the abdominal fat distribution may not have been estimated through BMI. Anthropometric indices (WC, WHR, WHtR) are used to estimate central obesity [25]. Indeed, the assessment of visceral obesity with new anthropometric indices (BAI, VAI, LAP, BRI, ABSI) compared to BMI could be a better evaluation in predicting either metabolic or cardiovascular complication [26,27]. Values of VAI > 1.675 may differentiate metabolically healthy PCOS women from unhealthy PCOS women [27]. The mentioned anthropometric indices are simple, cost-effective, noninvasive methods to evaluate and asses the cardiometabolic risk in PCOS women [26,27]. According to the higher rate of atherosclerosis and the prevalence of cardiovascular diseases in patients with PCOS, screening and management of CVD risk is crucial [15]. Moreover, values of Atherogenic Index Plasma (AIP) have been shown as promising for determining risk of MetS, hypertension, T2DM, and CVD, and other modern diseases. Both AIP and Castelli’s risk index-I (TC/HDL-C) and Castelli’s risk index-II (LDL-C/HDL-C) have also been measured in the study because the parameters of lipid profile may play an important role in metabolic diseases [28], including PCOS.

The aim of this study was to evaluate the relationship between fetuin-A concentration and anthropometric measurements and indices, body composition, and atherogenic indices, as well as glucose and lipid parameters, in patients with PCOS.

## 2. Methods

### 2.1. Study Population

This cross-sectional study consisted of 101 woman (aged 18–39; the median age was 25 years) who met the inclusion criteria for the study.

Criteria for selecting the subjects were as follows: diagnosed PCOS based on the Rotterdam criteria from 2003 (at least two of the following three criteria were met: oligo ovulation or anovulation, clinical and/or biochemical hyperandrogenism, polycystic ovaries visualized on ultrasound—12 or more follicles 2–9 mm in diameter in each ovary and/or increased ovary volume > 10 mL [1]); patient consent to the examination; aged between 18 and 40.

Exclusion criteria were as follows: the patient’s lack of consent to participate in the study; condition after implantation of cardiac pacemaker; pregnancy; lower limbs prostheses; foot dressings; no blood collected as part of routine or haemolysis of the blood; use of hormonal contraceptives, glicocorticosteroids, oral steroid medications or lipid-lowering drugs, or drugs that affect carbohydrate metabolism; previously diagnosed and treated diabetes mellitus; decompensated thyroid disorders; androgen excess disorders (congenital or late-onset Cushing’s disease/syndrome, congenital adrenal hyperplasia, androgen-secreting tumors, idiopathic hirsutism, hyperprolactinemia); depressive disorders and treatment of depression; diagnostic incomplete; re-hospitalization.

**Figure 1:**
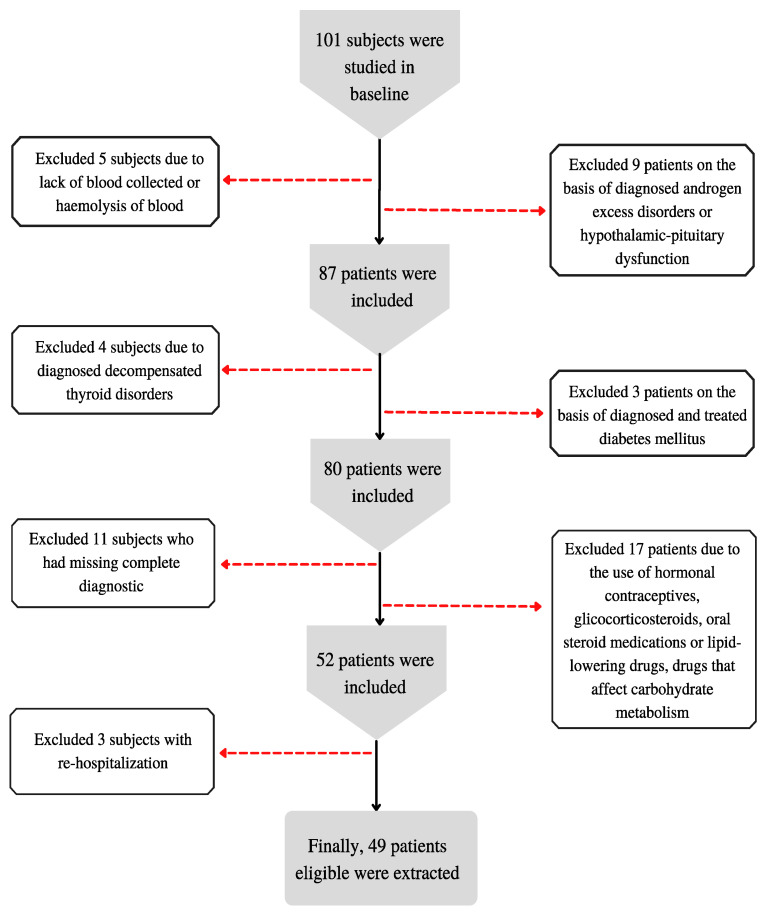
Patient qualification algorithm for the study.

A small sample was chosen because of the expected difficulty in obtaining women who met the inclusion criteria. The algorithm for qualifying patients for the study is presented in Figure 1.

The study conforms to the Declaration of Helsinki and was reviewed by the bioethics committee of the Medical University of Silesia (KNW/0022/KB1/143/15). Informed consent for data analysis was obtained from all participants.

### 2.2. Methods

Data for this study were collected between 2015 and 2018 at the Department of Endocrinology, Piekary Medical Centre, St. Luke’s Local Hospital in Piekary Śląskie, Poland.

Blood samples were taken from patients as part of routine measurements in the morning, before breakfast by qualified staff of the Department. One milliliter of blood, collected as part of routine tests, was preserved for further analysis and, after centrifugation, was frozen and stored at −70 degrees Celsius until the fetuin-A determinations were made. Other biochemical parameters (glucose levels, HbA1c, creatinine, GFR, total cholesterol, HDL, LDL, TG, CRP) and morphology (Hb, RBC, WBC, HCT, PLT, MCV, MCH, MCHC) were also determined and used for the calculation of three atherogenic indices: Castelli’s risk index-I (TC/HDL-C) and Castelli’s risk index-II (LDL-C/HDL-C), Atherogenic coefficient (AC) (TC-HDL-C/HDL-C), and Atherogenic index of plasma (AIP) (log(TG/HDL-C)).

Fasting anthropometric parameters were measured with the use of standard methods. These measurements included body weight (kg), height (cm), waist circumference (cm), and hip circumference (cm). Anthropometric indices calculated from these measurements included: BMI (Body Mass Index) = body weight (kg)/height (m^2^), WHR (Waist to Hip Ratio) = waist circumference (cm)/hip circumference (cm), WHtR (Waist to Height Ratio) = waist circumference (cm)/height (cm), BAI (Body Adiposity Index) = (hip circumference (cm)/height (m^1.5^)) − 18, VAI (Visceral Adiposity Index) = (waist circumference (cm)/(36.58 + [1.89 × BMI])) × (triglyceride concentration (mmol/L)/0.81) × (1.52/HDL concentration (mmol/L)), LAP (Lipid Accumulation Product) = (waist circumference (cm) − 58) × (triglyceride concentration (mmol/L)), BRI (Body Roundness Index) = 365.2 − 365.5 × √(1 − ((WC/2π)2)/[(0.5 × height)]^2^), ABSI (A Body Shape Index) = WC(m)/((BMI)^2/3^) × (height (m)^1/2^). The ABSI was also converted to z-scores, which include mortality risk in correlation with age and gender [26,29,30,31].

Body composition analysis was performed using the TANITA BC 420 MA analyzer (TANITA, Japan) with a certificate MDD 93/42 EEC for medical devices.

The tested measurements and anthropometric measurements, cut-off values, and standards are presented in Table 1.

Criteria for diagnosing hypertension, dyslipidemia (hypercholesterolemia, hypertriglyceridemia, mixed hyperlipidemia) [36], impaired fasting glucose, impaired glucose tolerance, diabetes mellitus, insulin resistance [37], and hypothyroids were as follows: hypertension: ≥140/90 without diagnosed hypertension or diagnosed hypertension, hypercholesterolemia: TC ≥ 4.921 mmol/L, hypertriglyceridemia: TG ≥ 1.694 mmol/L, mixed, hyperlipidemia: TC ≥ 4.921 mmol/L and TG ≥ 1.694 mmol/L, impaired fasting glucose 100–125 mg/dL, impaired glucose tolerance: at 120 min of OGTT blood glucose 140–199 mg/dL, diabetes mellitus—one of the following criteria: (1). symptoms of hyperglycaemia and random glycemia > 200 mg/dL, (2). two times fasting blood glucose ≥ 126 mg/dL, (3). 120-min glycemia OGTT ≥ 200 mg/dL, insulin resistance—as a limit value is HOMA-IR > 2, hypertension: TSH > 4.0 µIU/mL, fT3 < 3.0 pmol/L, fT4 < 10 pmol/L.

### 2.3. Laboratory Measurements

Fasting fetuin-A concentrations were determined in duplicates using the ELISA method. The BioVendor kit (Human Fetuin-A Elisa BioVendor, Czech Republic) was utilized. The intra-assay and inter-assay coefficient of variation were 2.9% and 4.7%, respectively. The tests were performed in accordance with the manufacturer’s instructions.

The value of the HOMA-IR (Homeostatic model assessment) index was calculated using the following formula:

HOMA-IR = fasting insulinemia (mU/mL) × fasting glycemia (mmol/L)/22.5.

### 2.4. Statistical Analysis

Statistical analysis was performed using the STATISTICA 13 PL software (Tulsa, OK, USA).

Continuous variables are expressed as means ± standard deviations (for normally distributed data) or median and interquartile range (for nonparametric data). The Shapiro–Wilk test was used to test the distribution. Normally distributed data were compared using the Student’s *t*-test, while nonparametric data were compared using the Mann–Whitney U test. A correlation between variables was evaluated using Pearson’s correlation coefficient (normal distribution) and Spearman’s rank correlation coefficient (non-normal distribution). A *p* value of less than 0.05 was considered significant.

## 3. Results

### Characteristics of the Study Group

A total of 49 women with PCOS diagnosis were included in the study. The median age of women was 25.00 (22.00–29.00) years. Baseline clinical and laboratory characteristic as well as anthropometric parameters and body composition of the study group are shown in Table 2 and Table 3.

Obesity was diagnosed in 18 patients (36.7%) based on BMI index and 7 patients (14.3%) based on BAI index. A significantly increased risk of metabolic complications was observed among 26 patients (53.1%) based on waist circumferences. Based on VAI index, risk of metabolic disease was observed among 17 women (34.7%).

Dyslipidemia, such as hypercholesterolemia, hypertriglyceridemia, and mixed hyperlipidemia was detected among 14 patients (28.6%), and insulin resistance was observed among 29 (59.2%). More information about risk of health and health conditions in the analyzed group are presented in Table 4.

There was an observed positive correlation between fetuin-A and total cholesterol (R = 0.302, *p* = 0.035). No significant correlation was found between fetuin-A concentration and the remaining laboratory results, nor with the anthropometric measurements/indices or the body composition analysis (Table 5).

There is no significant difference in mean concentration of fetuin-A among the groups of women regarding anthropometric parameters and indices and health status (Table 6).

## 4. Discussion

Overweight and obesity affect a large number of women (38–88%) suffering from PCOS. Therefore, it is important to determine the risk factors and complications related to overweight and obesity in women with this disorder. All of the above affect the clinical state and endocrine function in these women [38]. Our study is the first to assess the link between a significant amount of anthropometric measurements, indices, and body compositions, as well as atherogenic indices and fetuin-A concentrations in PCOS patients. Ix et al. focused on elderly patients (70–79 years of age) and showed a relationship between fetuin-A concentration and increased visceral adipose tissue during five-year follow-up. However, no relationship was found with respect to other studied elements of body composition [39]. Brix et al. examined patients with morbid obesity and found that fetuin-A concentration decreased in patients who lost weight after bariatric surgery [40]. Kozakowski et al. examined 40 PCOS women (26 lean and 15 obese) [41]. Based on BMI, no significant differences in fetuin-A concentration were found between the groups of obese and lean PCOS patients. Similarly, we did not find any relationship between the concentration of fetuin-A and body composition parameters, anthropometric parameters, and anthropometric indices. However, no previous study has investigated a correlation between anthropometric indices (VAI, BAI, LAP, BRI, ABSI, ABSI z-core) and serum of fetuin-A concentration in woman with PCOS. Moreover, to date only a few studies have used AIP indicators in woman with PCOS to analyze the risk of cardiometabolic disease. We found one study that examined the values of the HOMA-IR, Castelli Index, and AIP in PCOS [42]. Nawrocka-Rutkowska et al. highlights that the Castelli index and AIP are useful additional parameters to determine the risk of cardiometabolic disease in PCOS patients, especially with insulin resistance. Similarly, Kheirollahi et al. found that the use of indices (TG/HDL-C), Castelli index-I (TC/HDL-C), and TG is recommended in the assessment of insulin resistance risk among Iranian woman with PCOS [43].

IR is highly prevalent in PCOS (50–70%) [19]. By exerting the effect on ovarian androgen synthesis and decreased synthesis of sex hormone binding globulin (SHBG), insulin resistance may be responsible for the development of hyperandrogenism [44]. Fetuin-A, as a physiological inhibitor of insulin receptor tyrosine kinase, aggravates the risk of IR and type 2 diabetes [21]. Aroner et al. studied 455 patients with diabetes mellitus and 1957 healthy persons aged between 45 and 84. They found a strong correlation between higher concentrations of fetuin-A and the risk of diabetes mellitus in women. Regarding men, the correlation was not as strong as in women [45]. Similar results were obtained by Iyidir et al. in relation to gestational diabetes mellitus. The study group consisted of 26 pregnant women with gestational diabetes mellitus and 24 healthy pregnant women. Fetuin-A concentration were also evaluated in 18 women with gestational diabetes mellitus after delivery. Women with gestational diabetes were reported to have higher fetuin-A concentrations when compared to women after delivery [46]. Nevertheless, when considering patients with PCOS, the importance of fetuin-A in the pathogenesis of insulin resistance and dyslipidemia is not clear. In a study by Enli et al., 22 PCOS women aged 16–36 and 21 healthy women aged 18–36 were examined. PCOS women had significantly higher fetuin-A concentrations in comparison to healthy women. A positive correlation was found between fetuin-A concentration and insulin concentration and HOMA-IR index and FAI index [23]. Contrary to this, we did not find similar correlations in our study. Abali et al. [24] examined 35 PCOS women and 37 healthy women. As a result, no significant correlation was noted between fasting glucose, HOMA-IR, HBA1C, and fetuin-A concentrations in either study groups; however, a significant correlation was observed between fetuin-A concentrations and fasting insulin in healthy women. There was a significant difference in the concentration of fetuin-A between the study groups, which was also reported by Enli et al. [23]. In a study by Gulhan et al., there was no difference in fetuin-A concentration in the studied groups (44 women with PCOS aged 17–36 and 44 controls aged 18–38). However, a correlation between fetuin-A concentration and glycemia in PCOS patients was found [25]. We did not find any correlation between fetuin-A concentration and HOMA-IR, fasting insulin, fasting glucose, HbA1C, or glucose tolerance test results.

In addition to symptoms related to PCOS, they previously described diabetes mellitus, obesity, or insulin resistance; there are also others components (e.g., hypertension and dyslipidemia) that may increase the risk of cardiovascular disorders in PCOS. Some of them are coincident with metabolic syndrome components (central obesity, hypertriglyceridemia, decreased HDL cholesterol, hypertension, and abnormal glucose concentration). Therefore, PCOS patients also have an increased risk of metabolic syndrome [12,47]. We demonstrated that total cholesterol increased with the elevation of the fetuin-A concentration. Similar results were reported by Abali et al., who found a significant relationship between fetuin-A concentration and total cholesterol, LDL cholesterol, and triglyceride concentration, but no correlation between fetuin-A concentration and other parameters (e.g., BMI, HDL, fasting glucose, fasting insulin, HOMA-IR, or HBA1C) [24]. There is lack of correlation between fetuin-A and IR and BMI, but the positive correlation with TC remains unexplained. The hypothesis led by Ix et al. suggests that fetuin-A, by acting as a physiological tyrosine kinase inhibitor, can lead to hyperlipidemia through decreased insulin activity, resulting in increased lipolysis in the adipose tissue. By this mechanism the free fatty acids may be effluxed [24,48]. Low fetuin-A concentrations may predict cardiovascular risk. Moreover, fetuin-A may be a potent inhibitor in vascular calcification. It is possible that the upleveled calcification may shift the improvements in dyslipidemia [48]. Due to its possible complex explanation and underlying mechanism, the clinical relevance of the correlation between fetuin-A levels and its influential part on total cholesterol need to be confirmed in future studies.

## 5. Study Strength and Limitations

The study has a few limitations. Firstly, the study group was small, rendering not significant. However, the large number of exclusion criteria resulted in a very homogeneous group. Notwithstanding, studies on fetuin-A all had a similar number of participants to our study, from 11 patients with PCOS to 88 patients. The wide spectrum of PCOS clinical presentations may have various outcomes. In reviewing the literature, no data were found on the association between fetuin-A concentration and anthropometric indices (VAI, BAI, LAP, ABSI, ABSI z-core). This study provided an important opportunity to advance the understanding of fetuin-A concentrations and body composition, anthropometric indices, and metabolic disorders in woman with PCOS. Future studies that take these variables into account will need to be undertaken. More information on the relationship between fetuin-A concentrations and anthropometric indices would help us to establish a greater degree of accuracy in this matter.

## 6. Conclusions

Our study evaluated relationship between fetuin-A concentration with other metabolic parameters and indices in women with PCOS. We found that fetuin-A correlates with hypercholesterolemia. No significant correlation was found between fetuin-A concentration and the anthropometric measurements/indices, atherogenic indices, glucose parameter, or the body composition analysis. Based on our results, fetuin-A would not be a possible marker for PCOS women to evaluate cardiometabolic risk, especially glucose parameters and anthropometric parameters/indices. The effects of concentration of fetuin-A and anthropometric indices (BAI, VAI, LAP, ABSI, ABSI z-core) in woman with PCOS have not been closely examined before. It is necessary to conduct further research regarding the relationship between fetuin-A concentrations and body composition, anthropometric indices, and metabolic disorders in women with PCOS.

## Figures and Tables

**Table 1:** Interpretation of anthropometric parameters and indices.

Tested Measurements and Anthropometric Indicators	Standards	Interpretation of Standards	References
WC (cm)	>88 cm	significantly increased risk of metabolic complications	[32]
BMI Index (kg/m^2^)	<18.5 kg/m^2^	underweight	[33]
	18.5–24.9 kg/m^2^	normal body weight	
	25.0–29.9 kg/m^2^	overweight	
	30.0–34.9 kg/m^2^	I obesity	
	35.0–39.9 kg/m^2^	II obesity	
	≥40.0 kg/m^2^	III obesity	
WHR	≥0.85	increasing the risk of metabolic complications	[32]
WHtR	≥0.5	abdominal obesity	[32]
		increased risk	
		cardiovascular diseases	
		and diabetes	
BAI [%]	<21%	underweight	[34]
	21–33%	standard	
	>33%	overweight	
	>39%	obesity	
VAI	>1.675	risk of metabolic diseases	[27]
LAP	<41.30	discrimination of prediabetes/diabetes	[35]
BRI	<4.910	risk of metabolic diseases	[26]
ABSI	<0.076	risk of diabetes and CVD	[26,29]
ABSI z-score	<−0.868	mortality risk—very low	[29,30,31]
	−0.868 and −0.272	mortality risk—low	
	−0.272 and +0.229	mortality risk—average	
	0.229 and 0.798	mortality risk—high	
	>0.798	mortality risk—very high	

WC, waist circumference; BMI, body mass index; WHR, waist to hip ratio; WHtR, waist to height ratio; BAI, body adiposity index; VAI, visceral adiposity index; LAP, lipid accumulation product; BRI, body roundness index; ABSI, a body shape index; CVD, cardiovascular diseases.

**Table 2:** Baseline clinical and laboratory characteristic.

Parameter	Total Group (*N*)
Age (years)	26.29 ± 5.97 (49)
Biochemical parameters	
Fetuin (µg/mL)	226.75 (201.16–289.71) (49)
TSH (uIU/mL)	2.19 ± 1.07 (49)
Fasting insulin (pmol/L)	77.13 (48.07–106.91) (49)
Fasting glucose (mmol/L)	5.04 ± 0.68 (49)
Glucose after 120 min (glucose tolerance test) (mmol/L)	6.47 (5.36–7.77) (40)
Glucose after 60 min (glucose tolerance test) (mmol/L)	8.01 ± 2.42 (39)
Insulin after 60 min (glucose tolerance test) (pmol/L)	703.15 (325.03–835.17) (36)
HbA1c (%)	5.16 ± 0.91 (25)
HOMA-IR index	2.65 (1.40–4.08) (49)
Total cholesterol (mmol/L)	4.94 ± 1.05 (49)
HDL cholesterol (mmol/L)	1.67 ± 0.49 (49)
LDL cholesterol (mmol/L)	2.41 (2.06–3.29) (49)
Triglycerides (mmol/L)	0.93 (0.67–1.63) (49)
CRP (mg/L)	14.29 (3.81–49.52) (47)
Atherogenic indices	
Castelli’s risk index-I	2.92 (2.23–3.87) (49)
Castelli’s risk index-II	1.65 (1.06–2.33) (49)
Atherogenic Index of plasma	−0.19 ± 0.33 (49)
Atherogenic coefficient	1.92 (1.23–2.87) (49)

TSH, thyroid-stimulating hormone; HOMA-IR, homeostatic model assessment; HBA1C, glycated hemoglobin (A1c); HDL cholesterol, high density lipoprotein; LDL cholesterol, low-density lipoprotein; ±SD, standard deviation; Q1, lower of quartile; Q3, upper of quartile.

**Table 3:** Anthropometric and body composition measurements.

Parameter	Total Group (*N*)
Body weight (kg)	77.42 ± 21.03 (49)
Height (cm)	164.91 ± 5.84 (49)
Waist circumference (cm)	91.11 ± 18.87 (49)
Hip circumference (cm)	108.12 ± 13.30 (49)
BMI index (kg/m^2^)	28.34 ± 7.13 (49)
WHR	0.84 ± 0.10 (49)
WHtR	0.55 ± 0.11 (49)
BAI (%)	33.04 ± 5.69 (49)
VAI	1.22 (0.64–2.29) (49)
LAP	36.16 (9.83–67.80) (49)
BRI	4.63 ± 2.38 (49)
ABSI	0.08 ± 0.01 (49)
Body composition	
Body fat mass (kg)	48.37 ± 6.13 (49)
Percent of body fat (%)	37.73 ± 10.26 (49)
Lean body mass (kg)	48.37 ± 6.13 (49)
Muscle mass (kg)	46.11 ± 6.15 (49)
Water content (kg)	34.76 ± 5.38 (49)
Water content (%)	46.45 ± 6.34 (49)

BMI, body mass index; WHR, waist to hip ratio; WHtR, waist to height ratio; BAI, body adiposity index; VAI, visceral adiposity index; LAP, lipid accumulation product; BRI, body roundness index; ABSI, body shape index; ±SD, standard deviation; Q1, lower of quartile; Q3, upper of quartile.

**Table 4:** Interpretation of anthropometric parameters and health status of the study group.

Weight status by BMI:	
Underweight, *n* (%)	1 (2.0)
Normal weight, *n* (%)	17 (34.7)
Overweight, *n* (%)	13 (26.5)
Obesity, *n* (%)	18 (36.7)
Weight status by BAI:	
Underweight, *n* (%)	0 (0.0)
Normal weight, *n* (%)	24 (49.0)
Overweight, *n* (%)	18 (36.7)
Obesity, *n* (%)	7 (14.3)
Waist circumference (WC):	
Significantly increased risk of metabolic complications, *n* (%)	26 (53.1)
WHR:	
Increasing the risk of metabolic complications, *n* (%)	22 (44.9)
WHtR:	
Abdominal obesity increased risk of cardiovascular diseases and diabetes, *n* (%)	30 (61.2)
VAI:	
Risk of metabolic diseases, *n* (%)	17 (34.7)
LAP:	
Discrimination of prediabetes/diabetes, *n* (%)	27 (55.10)
BRI:	
Discrimination of prediabetes/diabetes, *n* (%)	28 (57.14)
ABSI:	
Risk of diabetes and cardiovascular disease, *n* (%)	21 (42.9)
ABSI z-score:	
Mortality risk—very low, *n* (%)	14 (28.6)
Mortality risk—low, *n* (%)	7 (14.3)
Mortality risk—average, *n* (%)	12 (24.5)
Mortality risk—high, *n* (%)	6 (12.2)
Mortality risk—very high, *n* (%)	10 (20.4)
Hypertension, *n* (%)	4 (8.2)
Dyslipidemia:	
Hypercholesterolemia, *n* (%)	10 (20.4)
Hypertriglyceridemia, *n* (%)	2 (4.1)
Mixed hyperlipidemia, *n* (%)	2 (4.1)
Impaired fasting glucose, *n* (%)	5 (10.2)
Impaired glucose tolerance, *n* (%)	9 (18.4)
Diabetes mellitus, *n* (%)	3 (6.1)
Hypothyroidism, *n* (%)	23 (46.9)
Insulin resistance, *n* (%)	29 (59.2)

BMI, body mass index; WHR, waist to hip ratio; WHtR, waist to height ratio; BAI, body adiposity index; VAI, visceral adiposity index; LAP, lipid accumulation product; BRI, body roundness index; ABSI, body shape index.

**Table 5:** Correlation between fetuin-A and selected anthropometric and biochemical parameters.

	*N*	R	*p*-Value
Body weight (kg)	49	−0.10	0.48
Waist circumference (cm)	49	−0.06	0.66
Hip circumference (cm)	49	−0.07	0.61
BMI index (kg/m^2^)	49	−0.06	0.65
WHR	49	0.01	0.93
WHtR	49	−0.04	0.78
Percent of body fat (%)	49	−0.20	0.17
BAI (%)	49	−0.01	0.90
VAI	49	0.08	0.57
LAP	49	0.08	0.58
BRI	49	−0.04	0.78
ABSI	49	0.03	0.83
Castelli’s risk index-I	49	0.04	0.75
Castelli’s risk index-II	49	0.03	0.82
Atherogenic Index of plasma	49	0.07	0.62
Atherogenic coefficient	49	0.04	0.75
Fasting insulin (pmol/L)	49	−0.08	0.56
Fasting glucose (mmol/L)	49	−0.11	0.42
Glucose after 120 min (mmol/L) (glucose tolerance test)	40	0.04	0.80
Glucose after 60 min (mmol/L) (glucose tolerance test)	39	0.08	0.62
Insulin after 60 min (pmol/L) (glucose tolerance test)	36	−0.06	0.70
HbA1c (%)	25	−0.13	0.52
HOMA-IR index	49	0.09	0.50
Total cholesterol (mmol/L)	49	0.30	0.03
HDL cholesterol (mmol/L)	49	0.09	0.53
LDL cholesterol (mmol/L)	49	0.14	0.30
Triglycerides (mmol/L)	49	0.15	0.29

BMI, body mass index; WHR, waist to hip ratio; WHtR, waist to height ratio; BAI, body adiposity index; VAI, visceral adiposity index; LAP, lipid accumulation product; BRI, body roundness index; ABSI, body shape index; HOMA-IR, homeostatic model assessment; HBA1c, glycated hemoglobin (A1c); HDL cholesterol, high density lipoprotein; LDL cholesterol, low-density lipoprotein.

**Table 6:** Concentration of fetuin-A according to anthropometric parameters and indices and health status.

		Concentration of Fetuin-A [µg/mL]		
		*N*	Mean ± SD	*p*-Value
BMI	Normal weight	17	249.24 ± 55.87	0.58
	Excess weight (overweight + obesity)	31	246.73 ± 63.28	
BAI	Normal weight	24	238.03 ± 53.09	0.36
	Excess weight (overweight + obesity)	25	259.82 ± 66.14	
WC	≤88 cm	23	246.47 ± 61.57	0.81
	>88 cm	26	251.51 ± 60.63	
WHR	WHR < 0.85	27	250.36 ± 60.18	0.75
	WHR ≥ 0.85	22	247.66 ± 62.24	
WHtR	WHtR < 0.5	19	239.52 ± 58.23	0.37
	WHtR ≥ 0.5	30	255.24 ± 62.06	
VAI	VAI ≤ 1.675	32	244.16 ± 58.60	0.51
	VAI > 1.675	17	258.54 ± 64.65	
LAP	LAP < 41.30	27	238.99 ± 56.73	0.24
	LAP ≥ 41.30	22	261.62 ± 63.89	
BRI	BRI < 4.910	28	251.97 ± 60.12	0.63
	BRI ≥ 4.910	21	245.39 ± 62.25	
ABSI	ABSI < 0.076	21	257.6 ± 68.8	0.50
	ABSI ≥ 0.076	28	242.8 ± 53.9	
ABSI z-score	ABSI <−0.868	14	251.82 ± 66.34	0.89
	ABSI −0.868 and −0.272	7	269.11 ± 77.39	
	ABSI −0.272 and +0.229	12	236.84 ± 46.87	
	ABSI 0.229 and 0.798	6	237.30 ± 62.74	
	ABSI > 0.798	10	253.32 ± 60.46	
HOMA-IR	Lack of insulin resistance	20	252.48 ± 52.70	0.51
	Insulin resistance (EGIR—The European Group for the Study of Insulin Resistance)	29	246.85 ± 66.67	

HOMA-IR, homeostatic model assessment, WC, waist circumference, BMI, body mass index; WHR, waist to hip ratio; WHtR, waist to height ratio; BAI, body adiposity index; VAI, visceral adiposity index; LAP, lipid accumulation product; BRI, body roundness index; ABSI, body shape index.

## Data Availability

All data are available via email from justyna.nowak@sum.edu.pl.
